# Patients’ Satisfaction With the Quality of Services at Primary Healthcare Centers in Saudi Arabia

**DOI:** 10.7759/cureus.45066

**Published:** 2023-09-11

**Authors:** Shahad M Alhajri, Najla M Aljehani, Mervat M El Dalatony, Saleh S Alsuwayt, Turki M Alhumaidany, Mohammed S Aldossary

**Affiliations:** 1 Research and Studies General Department, Ministry of Health, Riyadh, SAU; 2 Public Health Department, College of Health Sciences, The Saudi Electronic University, Riyadh, SAU; 3 Public Health and Community Medicine Department, Faculty of Medicine, Menoufia University, Shebin Elkom, EGY; 4 Family Medicine Department, King Saud Medical City, Ministry of Health, Riyadh, SAU; 5 Patient Experience Department, Ministry of Health, Riyadh, SAU

**Keywords:** primary healthcare centers, saudi arabia, quality of healthcare, patient-centered care, patient satisfaction, patient experience

## Abstract

Background: Patients’ satisfaction is an essential indicator used to measure the quality of healthcare services. The evaluation of patients’ satisfaction with primary healthcare center (PHC) visits is therefore essential when it comes to assessing the quality of healthcare services provided.

Aim: To assess patients’ overall satisfaction with the quality of services provided at PHCs in different regions of Saudi Arabia.

Methods: A retrospective cross-sectional study was conducted using secondary data collected from 2,390 PHCs in different regions of Saudi Arabia. Data were collected by the Patient Experience Measurement Program in the Ministry of Health (MOH) using the Press Ganey Survey from January 2022 to December 2022. The Press Ganey Survey is a standardized tool used by the Saudi MOH to assess patients’ satisfaction with the health services provided in different specialties and healthcare facilities. The assessment tool (Press Ganey survey) involves six domains (Access, Moving through, Nurse, Care provider, Personal issue, and Overall assessment). The data were tabulated and analyzed using the Statistical Package for the Social Sciences (SPSS).

Results: In total, there were 536,406 study participants, with their mean age being 28.7±21.1. The overall patients’ satisfaction score was 4.2 out of 5 (83.8%). Male participants reported more satisfaction with PHC services than did females (86.6%, 81%), respectively. The domain with the highest patients’ satisfaction scores was that of the Care provider (84.8%), while the domain with the lowest rating was Moving through (82.8%).

Conclusion: This baseline study found that patients were generally satisfied, with the highest levels of satisfaction with their care providers, highlighting the crucial role of professionals in the patient experience. Further research is recommended to give a more in-depth analysis and thus highlight additional correlating and predicted elements of patients' satisfaction with PHC treatments in Saudi Arabia.

## Introduction

The concept of healthcare quality is integral when it comes to programs for quality assurance and development in the healthcare sector [1]. Quality in the healthcare sector has recently gained momentum as a result of the creation of quality assurance, initiatives for quality improvement, and patient agendas [2]. Patients' satisfaction has long been considered a critical factor in determining the health outcome and quality level of healthcare [3], since it serves as a major indicator of the quality of healthcare services [4]. Previous studies showed that satisfied patients were more likely to establish positive relationships with the healthcare system, thus improving compliance and continuity of care and eventually contributing to better health outcomes [5]. Patients' satisfaction is considered an important indicator of how well health services are performing, as it can forecast both compliance and use. As a result, the restructuring of healthcare systems operating globally has focused on strategies for increasing patients' satisfaction.

The patient's initial interaction with the healthcare system is through primary healthcare. Indeed, the patient has interactions with primary healthcare centers (PHCs) in several domains, including, but not limited to, access to service, continuity of care, and communication with the provider [6]. Past studies indicated that patients’ satisfaction with their healthcare services is one of the main variables impacting healthcare outcomes and quality of service [4]. The evaluation of patients’ satisfaction with PHC visits is therefore essential when it comes to assessing the quality of care and indicating potential areas for expanding the PHCs’ scope of practice. Patients’ satisfaction is crucial because it identifies the strengths and weaknesses of healthcare, thus helping in improving the quality of treatment and future planning. Identifying detailed dimensions of patients’ satisfaction also aids in putting in place a framework designed to improve healthcare quality.

The Ministry of Health (MOH) Patient Experience Measurement Program is a National Transformation Program (NTP) that aims to enhance patients’ experience and create distinctive healthcare. The program surveys patients and their families after each visit so as to measure their satisfaction with MOH's health services and thus enhance quality. The program is implemented in many facilities in the MOH, such as hospitals, specialty centers, primary care centers, blood banks, smoke cessation centers, premarital screening centers, the 937-service center, and other therapy-providing institutions [7].

Although different studies have discussed the level of patients’ satisfaction in PHC settings in different regions of Saudi Arabia, to our knowledge, there is a lack of studies exploring the satisfaction levels in all MOH PHCs throughout the kingdom. Therefore, the aim of the current study is to assess patients’ satisfaction with the quality of healthcare services provided at PHCs across all regions of Saudi Arabia. The objective is to identify specific aspects of patient satisfaction with PHCs through their ratings of experience to improve healthcare quality and inform health services development.

## Materials and methods

The study was carried out to investigate patients' satisfaction with PHCs and, more specifically, to highlight areas for improvement in the healthcare system services provided by PHCs in Saudi Arabia.

This research was based on secondary data that were retrieved from the MOH Patient Experience Measurement Program, which contracted with an independent third party, Press Ganey. The Press Ganey has established its reputation as a world expert in assessing and enhancing patients’ experience. Using a third party improved the quality of the data, as it reduced bias during the process of data collection and analysis.

The MOH Patient Experience Measurement Program, which is responsible for patients’ experience in Saudi Arabia, collected the data related to patients’ experience through Press Ganey from 2,390 PHCs distributed throughout the Kingdom of Saudi Arabia.

Study participants were those patients who responded to the MOH patient experience online survey provided following their consent to participate. The survey was sent to all patients' mobile phones after each visit to any MOH PHC. The data were requested from the Patient Experience Measurement Program only for PHC visitors between January 1, 2022 and December 31, 2022. There were no expected risks to the participants in terms of data privacy and confidentiality, and no personal information was disclosed. In addition, the data used were securely stored on MOH servers and will be destroyed after a set period of time.

The survey consists of 23 questions grouped into six domains (Table 1): Access (three questions), Moving through (five questions), Nurse (three questions), Care provider (five questions), Personal issue (four questions), and Overall assessment (three questions). The rating was scored as follows: very poor (score =1), poor (2), fair (3), good (4), and very good (5).

**Table 1:** The six domains and related questions of the questionnaire.

Domain	Questions
Access	Ease of scheduling your appointment
	Ease of contacting the center (e.g., email, phone, web portal/application)
	Courtesy of staff in the file request area
Moving through	Ease of the file request process
	Degree to which you were informed about any delays
	How do you evaluate your wait time at the clinic?
	Comfort of the waiting area
	Bathroom cleanliness
Nurse	How well did the nurse listened to you?
	Concern the nurse showed for your problem
	Friendliness/courtesy of the nurse
Care provider	Concern the physician showed for your questions or worries
	Explanations the physician gave you about your problem or condition
	Physician’s efforts to include you in decisions about your treatment
	Physician’s discussion of any proposed treatments (options, risks, benefits, etc.)
	Likelihood of your recommending this physician to others
Personal issue	Our concern for your privacy
	How well did medical staff protect your safety (by sanitizing hands, wearing gloves, etc.)?
	Cleanliness of our clinics
	Parking
Overall assessment	How well did the staff work together to care for you?
	The likelihood of you recommending this center to others
	Overall rating of care received during your visits

Data analyses

The data collected were transferred to an Excel file (Microsoft® Excel®, Microsoft® Office 2019, Microsoft® Corp., Redmond, WA, United States), and then the data were statistically analyzed using IBM SPSS Statistics for Windows, Version 25.0 (Released 2017; IBM Corp., Armonk, New York, United States).

Descriptive statistics were generated for the demographic variables: region (five regions: Northern, Eastern, Western, Southern, and Central), gender (male, female), age (in years), and nationality (Saudi, Non-Saudi). The mean and standard deviation (SD) of the satisfaction scores were calculated for the six domains.

To compare the means of satisfaction between regions, the one-way ANOVA test was used. By utilizing the one-way ANOVA test, it was possible to compare the means across multiple groups and to ascertain whether there were any significant differences in the mean values of response variables for different categories.

To compare the means of satisfaction between the gender categories (male vs. female) and nationality (Saudi vs. non-Saudi), the t-test was used because these variables had two categories each. For all statistics, if the P-value was lower than 0.05, the result was deemed significant.

Ethical approval

In this study, all procedures followed the Helsinki Declaration, 2002, and we obtained ethical approval from the Central Institutional Review Board (H-01-R-009) in the MOH, Riyadh, Saudi Arabia (IRB log No: 23-10 M).

## Results

A total of 557,049 patients responded and submitted the survey. Only completed surveys were included (n=536,406) (96.3%), while incomplete surveys (3.7%) were excluded from the study.

Participants' demographics

Table 2 displays the demographic characteristics of the study participants. Among the participants, 51% were male and 49% were female; 93% were Saudi, while 7% were non-Saudi. The mean age of the participants was 28.7 (SD ±21.1).

**Table 2:** The demographic results of the participants (n=536,406).

Variable	Categories	Frequency (N)	%
Region	Northern	45,220	8.4
	Eastern	105,745	19.7
	Western	138,957	25.9
	Southern	102,363	19.1
	Central	144,121	26.9
Gender	Male	274,318	51.1
	Female	262,088	48.9
Nationality	Saudi	498,239	92.9
	Non-Saudi	38,167	7.1
Age	Median=30, mean=28.7, SD ±21.1		

Regarding the geographic distribution of the study participants, the Central region presented the highest percentage (27%) of the total participants, followed by the Western region (26%), the Eastern (20%), and then the Southern (19%); lastly, the Northern region presented 8%.

Satisfaction rate

The overall results of the satisfaction scores of the various domains are displayed in Table 3. The overall patients’ satisfaction score was around 4.2 out of 5 (83.8%, SD: 0.55). More people were confident and satisfied with services rendered by healthcare providers, as clearly shown by the fact that this domain had the highest mean satisfaction score, i.e., 4.24 (84.8%) with a SD of ±0.59, while the lowest score was that of the Moving through domain of the Press Ganey survey, which had an overall satisfaction rate of 82.8%.

**Table 3:** The overall results of the satisfaction scores of the six domains of the Press Ganey survey in PHC settings. SD: standard deviation; PHC: primary healthcare center.

Domain	Access	Moving through	Nurse	Care provider	Personal issue	Overall assessment	Overall patient satisfaction
Mean of satisfaction score (% of 5)	4.18 (83.6%)	4.14 (82.8%)	4.19 (83.8%)	4.24 (84.8%)	4.21 (84.2%)	4.16 (83.2%)	4.19 (83.8%)
SD	0.43	0.58	0.59	0.59	0.50	0.63	0.55

The mean patients’ satisfaction scores in relation to demographic regions are displayed in Table 4. The highest score was seen in the Southern region (4.44; 88.8%), while the lowest was in the Western region (4.06; 81.1%).

**Table 4:** Comparison of the mean score of satisfaction per region (one-way ANOVA test). P-value is considered significant at the level of <0.05. SD: standard deviation.

Variable	Categories	Mean of satisfaction score (% of 5)	SD	P-value
Region	Northern	4.19 (83.7)	0.53	0.001
	Eastern	4.24 (84.8)	0.48	
	Western	4.06 (81.1)	0.57	
	Southern	4.44 (88.8)	0.61	
	Central	4.11 (82.3)	0.51	

When it came to gender differences, higher satisfaction was reported among males compared to their female counterparts. Furthermore, the level of satisfaction among the research participants was different based on their nationality, with non-Saudi respondents reporting higher levels of satisfaction compared to their Saudi counterparts as presented in Table 5. All comparisons were statistically significant (P<0.05).

**Table 5:** Comparison of satisfaction according to the participant’s gender and nationality (t-test). P-value is considered significant at the level <0.05. SD: standard deviation.

Variable	Categories	Mean of satisfaction score (% of 5)	SD	t	95% CI	P-value
Gender	Male	4.33 (86.6)	0.50	205.01	0.277 to 0.283	0.001
	Female	4.05 (81)	0.58			
Nationality	Saudi	4.17 (83.4)	0.56	112.97	-0.305 to -0.295	0.001
	Non-Saudi	4.47 (89.4)	0.54			

## Discussion

The United Nations placed health, the timeless topic of humanity, at the center of the global sustainable development goals (SDGs) in December 2015. In 2018, the WHO updated its worldwide reference list of 100 core health indicators and included patient satisfaction as an indicator that can be used to assess the quality and safety of care in health systems [8]. Hence, patients’ satisfaction is a crucial indicator of the quality of healthcare since it reveals how well the provider meets clients' expectations. Additionally, patients’ satisfaction acts as a major factor in determining their point of view regarding their healthcare experience [2]. Without the customer's voice, no one can define quality. The most recent measurements that look at patient experiences actually represent experiences of quality.

Alghamdi et al. suggested conducting routine evaluations of patients’ satisfaction with PHC services so as to improve the quality of care provided. Current studies in Saudi Arabia have examined patients’ satisfaction with PHC services provided in different regions of the country, which ranged from 78% to 83%, but few have covered all the regions of Saudi Arabia [9].

A total number of 536,406 patients and patients’ guardians were included in the current study, which highlighted and investigated the variables influencing those participants’ satisfaction. The findings of the current study demonstrated that most of the participants were satisfied with the provided PHC services, with a satisfaction rate of 84%. The healthcare provider acts as the cornerstone of this high satisfaction level, as our study revealed a high level of satisfaction with the services offered by healthcare providers, followed by nurses. These results coincide with those of Owaidh et al. [10], who reported in their study that was conducted in Al Baha region of Saudi Arabia, a high rate of patients’ satisfaction among those who visited PHCs. In the aforementioned study, patients were 90% satisfied with the care they received from doctors, 83% with the nurses' services, and 80% with the facility's cleanliness, tranquility, and layout. According to Senitan et al. [11], an integral part of patients’ satisfaction is physician communication, which can have further effects on such satisfaction. Similar research has found that interactions between the physician and the patient are critical [12]. These results highlight the influential and significant role that healthcare providers have on the overall patients’ experience.

The results of the current study reported similar levels of patients’ satisfaction to what was reported by Mohamed et al. [3] in Al Majmaah city (82%), which is located in the Central region of Saudi Arabia. Moreover, our study revealed that the main pillar of these results was attributed to the Care provider, followed by Personal issue (patient privacy, cleanliness of clinic), and then the nurse domain. However, Mohamed et al. [3] stated that their patients’ satisfaction was due to the cleanliness of centers, staff competence, respect, and excellent handling. Another study, carried out in the Al Hasa region of Saudi Arabia, corroborates the findings of the current work, as the authors reported an 86% level of patients' satisfaction with the PHC services provided [6].

In addition to the above, a study conducted by Gao et al. [13] in China, with the participation of 1,138 patients ‎attending 728 PHC centers distributed over 31 counties, reported a satisfaction level of around 68%, ‎which was highly exceeded by our study results. However, with a finding similar to ours, Gao et ‎al. [13] indicated that patients’ satisfaction is significantly correlated with criteria related to ‎healthcare providers, such as professional level, the consultation process, accessibility, and ‎convenience.

Two of the key objectives of Saudi Vision 2030 are facilitating access to healthcare services and improving the quality and efficiency of health services. In line with Saudi Vision 2030, the NTP 2020 reform strategy emphasizes the necessity of closing the gap when it comes to healthcare access, with a focus on Universal Health Coverage (UHC). The UHC is a global initiative that aims at ensuring that all people have access to the health services they require. The services can range from public health services designed to promote better health (such as anti-tobacco information campaigns and taxes) or prevent illness (such as vaccinations) to providing treatment, rehabilitation, and palliative care (such as end-of-life care). It also aims at ensuring that users are not exposed to finite resources [14].

Moreover, the NTP 2020 aspires to establish primary healthcare as a "patient-centered model of care" and to establish "management units," introducing the concept of health service corporatization [15]. Indeed, Al Khashan et al. [15] stated that, by the middle of 2019, the reform had increased PHC visits, patients’ satisfaction, coverage of rural communities, and the rate at which people were being screened for prevalent chronic diseases. Hence, the result of this work seems very logical when considering the great efforts made by the Saudi MOH to improve healthcare provider’s professional levels through professional training, continuous medical education activities, and competency in PHC practice and services.

In the current study, the highest satisfaction level was reported in the Southern region, followed by the Eastern region, while the lowest level was in the Western region. This may be due to a general lack of health literacy (HL) or a greater reluctance to seek medical attention among patients in different geographic areas. Indeed, the aforementioned was reported by Almubark et al. [16], who revealed that 46% of their representative sample of the population in Saudi Arabia had low HL.

When it came to gender differences, male participants were significantly more satisfied with the service level provided at the PHC than were female participants. This is in concordance with the results of Owaidh et al. [10], who carried out their study at an emergency department in central Saudi Arabia and found that males had higher satisfaction rates than did females. Contrary to these findings, Abolfotouh et al. [17], whose study was carried out at an emergency care center, concluded that lower satisfaction with the emergency department visit was significantly associated with the male gender.

Concerning participants’ nationality, non-Saudi residents were significantly more satisfied with the services provided for them at the PHCs throughout the kingdom. This finding might be explained by their gratitude and loyalty to the kingdom, as all PHC services are provided to them for free. Providing a health service free of charge is an important part of the definition issued by the WHO, known as “Universal Health Coverage” [14].

Study strengths and limitations

The current research involved a relatively large number of participants, so it is deemed nationally representative. The study participants are of both genders, from all age groups, and are distributed throughout all regions of Saudi Arabia. Hence, the study results can be generalized. In addition, our results reflect the realistic impression of health service recipients in Saudi Arabia, which acts as a cornerstone in the process of improving healthcare service quality. However, the study was prone to a few limitations. One of the limitations was the lack of open-ended questions, which would have provided a full understanding of the participants’ perspectives. Added to this were the large amount of data lost due to incomplete questionnaires (n=20,643).

## Conclusions

In conclusion, our study found that, in Saudi Arabia, patients were satisfied with their experience at PHCs. Our study highlighted the importance of the feedback regarding patients’ satisfaction with the service they received as a guide to improve the quality of healthcare. We recommend further research to provide more in-depth analysis and thus highlight more correlated and predictive factors of patients’ satisfaction with PHC services in Saudi Arabia, particularly since the current study is only descriptive in nature.
